# Production of Putative Diterpene Carboxylic Acid Intermediates of Triptolide in Yeast

**DOI:** 10.3390/molecules22060981

**Published:** 2017-06-13

**Authors:** Victor Forman, Roberta Callari, Christophe Folly, Harald Heider, Björn Hamberger

**Affiliations:** 1 Evolva A/S, Lersø Park Allé 42-44, Copenhagen Ø DK-2100, Denmark; victorf@evolva.com; 2 Evolva SA, Duggingerstrasse 23, Reinach CH-4153, Switzerland; robertac@evolva.com (R.C.); christophef@evolva.com (C.F.); haraldh@evolva.com (H.H.); 3 Department of Biochemistry and Molecular Biology, Michigan State University, 603 Wilson Road, East Lansing, MI 48824, USA

**Keywords:** triptolide, dehydroabietic acid, miltiradienic acid, miltiradiene, dehydroabietadiene, medicinal diterpenes, *Saccharomyces cerevisiae*, *Nicotiana benthamiana*, *Tripterygium wilfordii*

## Abstract

The development of medical applications exploiting the broad bioactivities of the diterpene therapeutic triptolide from *Tripterygium wilfordii* is limited by low extraction yields from the native plant. Furthermore, the extraordinarily high structural complexity prevents an economically attractive enantioselective total synthesis. An alternative production route of triptolide through engineered *Saccharomyces cerevisiae* (yeast) could provide a sustainable source of triptolide. A potential intermediate in the unknown biosynthetic route to triptolide is the diterpene dehydroabietic acid. Here, we report a biosynthetic route to dehydroabietic acid by transient expression of enzymes from *T. wilfordii* and Sitka spruce (*Picea sitchensis*) in *Nicotiana benthamiana*. The combination of diterpene synthases *Tw*TPS9, *Tw*TPS27, and cytochromes P450 *Ps*CYP720B4 yielded dehydroabietic acid and a novel analog, tentatively identified as ‘miltiradienic acid’. This biosynthetic pathway was reassembled in a yeast strain engineered for increased yields of the pathway intermediates, the diterpene olefins miltiradiene and dehydroabietadiene. Introduction in that strain of *Ps*CYP720B4 in combination with two alternative NADPH-dependent cytochrome P450 reductases resulted in scalable in vivo production of dehydroabietic acid and its analog from glucose. Approaching future elucidation of the remaining biosynthetic steps to triptolide, our findings may provide an independent platform for testing of additional recombinant candidate genes, and ultimately pave the way to biotechnological production of the high value diterpenoid therapeutic.

## 1. Introduction

Specialized (formerly termed secondary) plant metabolites have been exploited throughout history as sources of beneficial natural products with wide ranges of applications. With around 7000 known structures, labdane-type diterpenes—characterized by a bicyclic decalin core—represent a major fraction of known diterpenes. This class has attracted much attention due to an increasing number of applications in industries spanning from perfumes to complex medicinal compounds [1,2,3]. Triptolide is a therapeutic, labdane-type diterpene that has been heavily investigated over the last four decades since its recognition as the main active principle in the Chinese medicinal herb *Tripterygium wilfordii* (Celastraceae), also known as the thunder god vine [4]. Triptolide is currently under investigation for anti-cancer activities [5,6], against inflammatory conditions [7], and Alzheimer’s disease [8] among other putative pharmaceutical applications. Availability of triptolide, which is isolated from *T. wilfordii* plants, is limited due to a content in the range of 6–16 ng·g^−1^ fresh weight [9]. An alternative route to access triptolide by formal chemical synthesis has been sought over the last three decades but remains economically infeasible at over 16 steps [10].

This work presents the proof-of-concept for production of a potential key intermediate in the biosynthesis of triptolide in engineered strains of baker’s yeast, *Saccharomyces cerevisiae*. Yeast has been shown to be a suitable biotechnological host for production of plant terpenes, exemplified by the semi-synthetic production of the antimalarial sesquiterpenoid artemisinin [11] and recently production of macrocyclic diterpenes [12] and cAMP booster forskolin [13]. Yet, such approaches require knowledge of the biosynthetic routes.

Plants produce diterpene hydrocarbons from the linear and achiral precursor (*E*,*E*,*E*,)-geranylgeranyl diphosphate (GGPP) by action of diterpene synthases (diTPSs) [14]. diTPSs can be classified depending on specific domains and their activities, i.e., class II diTPSs carry an aspartate-rich “DXDD” motif involved in cyclisation of GGPP into a cyclic diphosphate intermediate, such as the canonical *ent*-copalyl diphosphate, and precursor for plant gibberellin phytohormones [1,15]. Class I diTPSs contain a characteristic “DDXXD” motif responsible for the diphosphate cleavage and rearrangement of products into the specific diterpene backbones, with a reaction termination through proton abstraction or water quenching [1,15]. Products of plant diTPS enzymes are typically further functionalized into complex, biologically active compounds through oxidation by cytochrome P450-dependent monooxygenases (P450s). Resulting alcohol or carboxyl functions may enable further conjugation by various transferases [16,17,18,19].

**Figure 1:**
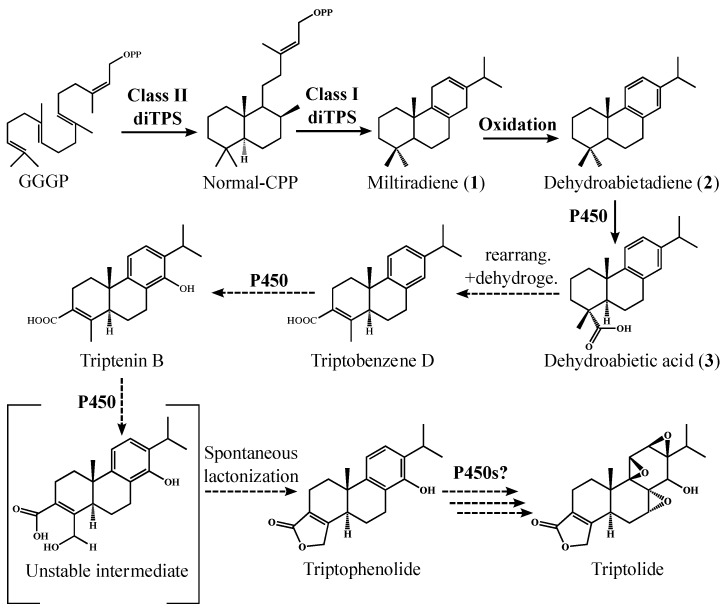
Proposed pathway to triptolide with a key intermediate, dehydroabietic acid. Dehydroabietic acid (**3**) is produced from dehydroabietadiene (**2**), a product of a pair of diTPS via copalyl diphosphate (normal-CPP) in normal, or (+) stereochemical configuration, to miltiradiene (**1**) that can undergo spontaneous aromatization into **2**. **2** is functionalized into **3** by CYP720B4, cytochrome P450. **3** possibly undergoes a carboxylic rearrangement and dehydrogenation to triptobenzene D as a subsequent step. The remaining steps in *T. wilfordii* towards triptolide could be catalyzed by other P450s. All diterpenoid intermediates in the proposed pathway have been reported in *T. wilfordii*, with exception of the unstable intermediate that undergoes spontaneous lactonization into triptophenolide.

The biosynthesis of triptolide has not been established, but the functional complement of diTPSs, expressed in the root of the plant has been discovered [20]. Specifically, the pair of *Tw*TPS9 and *Tw*TPS27 were found to catalyze the successive cyclization of GGPP to miltiradiene (**1**). Based on those findings and extensive metabolomic knowledge of reported diterpenes from *T. wilfordii* [21,22,23], a potential route is herein suggested (Figure 1). The pathway implies **1**, dehydroabietadiene (**2**) and dehydroabietic acid (**3**) as central key intermediates in the biosynthesis of triptolide. This route entails further carbon-rearrangement of the C-18 carboxylic group in **3**, dehydrogenation yielding triptobenzene D and lactonization of an unstable hydroxylated intermediate product of triptenin B. A simplified pathway towards triptolide has previously been proposed, also involving **3** as an intermediate [22]. A semi-synthetic conversion from **3** to triptolide was demonstrated earlier [24], practically mimicking the proposed biosynthetic route. Finally, detection of numerous putative intermediates of triptolide [25] lends further support for our hypothesis.

Our efforts therefore initially aimed at biotechnological production of **1** in yeast, using functionally established enzymes of *T. wilfordii*. The formation of **3** from **2** was earlier reported, catalyzed in the conifer Sitka spruce by *Ps*CYP720B4, member of the family of cytochrome P450 monooxygenases. *Ps*CYP720B4 was found in the CYP85 clan, as part of a largely expanded family with up to a dozen members in each conifer species investigated. Functionally, *Ps*CYP720B4 is involved in conifer defense related formation of diterpene resin acids. The conversion of a range of olefinic substrates follows a three-step oxidation, via a hydroxyl and carbonyl C-18, to the corresponding carboxylic acid [26]. While the biosynthetic route to the diterpene hydrocarbon backbone **2** in conifers remains elusive, in members of the Lamiaceae, **2** was suggested to be an non-enzymatic oxidation product of **1** (not reported in the investigated conifer systems) [19], and with that providing the missing link and a possible route of access to **3**.

Here we utilized transient expression in *Nicotiana benthamiana* (tobacco) to test and reconstruct a suitable biosynthetic route. This then guided engineering of yeast for production. Following initial detection of **1** and **2** formed by the pair of *T. wilfordii* diterpene synthases in yeast, the production levels were increased by synthetic expression optimization of the sequences. Heterologous co-expression of the similarly optimized P450 resulted in efficient conversion of **2** to the fully oxidized **3**. In addition, we observed formation of a likely structural analog, the novel C-18 carboxylic acid (**4**), plausibly derived from **1**. These findings pave the way for biotechnological production of bioactive diterpenes and may assist in the future in the full elucidation of the remaining triptolide pathway by providing a test-platform for candidate genes.

## 2. Results

### 2.1. Identification of a Biosynthetic Pathway to Dehydroabietic Acid by Transient Expression in Tobacco

Two diterpene synthases *Tw*TPS9 and *Tw*TPS27 from *T. wilfordii* were recently shown to afford miltiradiene (**1**), which, due to a nearly flat structure of the C-ring readily aromatizes to dehydroabietadiene (**2**) [20,27], and with that catalyze the committed first steps in our proposed route to triptolide. *Ps*CYP720B4 from Sitka spruce had previously been shown to catalyze formation of diterpene resin acids. Specifically, the enzyme was found most active with exogenously supplemented **2** over a range of diterpene olefins tested, yielding dehydroabietic acid (**3**) both in vitro and in vivo. We thus co-expressed *Tw*TPS9, *Tw*TPS27, and *Ps*CYP720B4 transiently in *N. benthamiana* to confirm a function of this combination of enzymes from divergent species (Table S1). The in planta yield of diterpenes was increased by co-expression of 1-Deoxy-d-xylulose 5-phosphate synthase (DXS) and a GGPP synthase, enzymes from the upstream 2-*C*-methyl-d-erythritol 4-phosphate pathway (MEP), to increase product formation to levels permitting purification and structural determination as earlier described [27].

**Figure 2:**
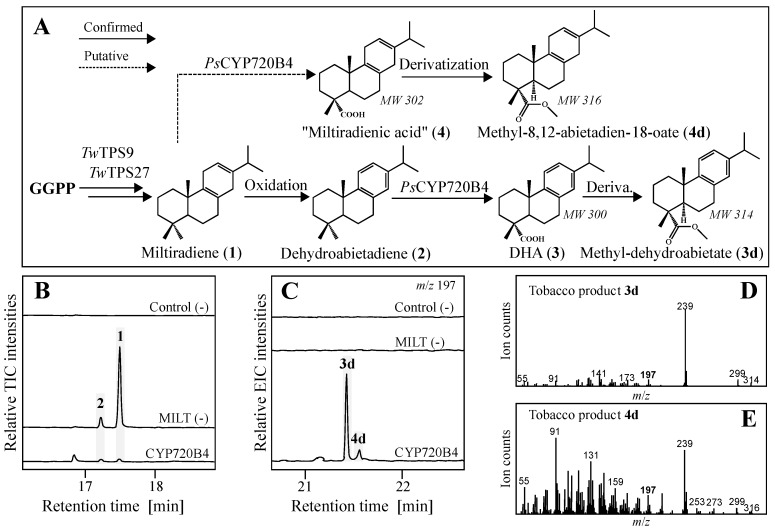
Identification of a dehydroabietic acid (**3**) pathway by transient expression in tobacco. (**A**) Transiently expressed genes with confirmed and putative diterpene products detected after derivatization; MW, molecular weight, corresponding to *m*/*z* for **4d** and **3d**; (**B**) Total ion chromatograms (TIC) of control plants without diTPS, plants expressing *Tw*TPS9 and *Tw*TPS27 (MILT (-)) and the addition of *Ps*CYP720B4; (**C**) Extracted ion chromatograms (EIC) of *m*/*z* 197, characteristic for both diterpene products, showing novel peaks **3d** and **4d** when *Ps*CYP720B4 was expressed in combination with diTPSs; (**D**) MS spectra of **3d** corresponding to derivatized methyl-ester of **3**; (**E**) MS spectra of **4d** corresponding to putative methyl-ester of miltiradienic acid (**4**).

Gas-chromatography mass spectrometry (GC-MS, Figure 2) revealed formation of the diterpenes **1** and, to a minor extent, **2** in plants transiently expressing the pair of diTPS. Co-expression with *Ps*CYP720B4 lead to near depletion of the diterpene olefins concomitant with efficient formation of new compounds (**3d** and **4d**), as identified in trimethylsilyl-diazomethane (TMDS) derivatized samples. The mass over charge ratio (*m*/*z*), retention times and fragmentation patterns were consistent with oxidation of the diterpenes into their corresponding carboxylates (Figure 2A), detected after derivatization to the methyl-ester of **3**, identified by comparison with previously reported MS spectra, and minor amounts of the putative methyl-abieta-8,12-dien-18-oate (**4d**), corresponding to **1** analogously oxidized at C-18. Thus, the combination *Tw*TPS9, *Tw*TPS27, and *Ps*CYP720B4 established an efficient, yet transient biosynthetic route towards **3** in tobacco, which encouraged engineering of yeast for heterologous production.

### 2.2. Production of Diterpene Backbones in Yeast

The biosynthetic pathway identified in tobacco was utilized for heterologous expression in yeast. Yields of diterpene olefins were optimized by heterologous expression of *Tw*TPS9 and *Tw*TPS27 in vivo in yeast as recombinant variants: diTPSs were codon optimized for expression in yeast, in full length (strain YS1); codon optimized and truncated (predicted chloroplast transit peptide sequences removed) diTPSs [27] to result in pseudomature forms (strain YS2); native coding sequence from *T. wilfordii* in full length (strain YS3) or truncated (strain YS4).

**Figure 3:**
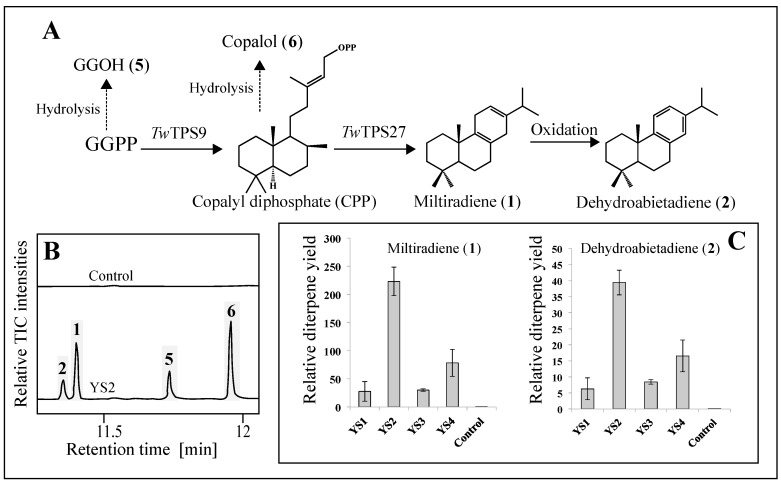
Production of diterpene olefins **1** and **2** in engineered yeast. (**A**) Pathway leading to dehydroabietadiene from GGPPS. Putative hydrolyzed products of GGPP into GGOH (**5**) and copalyl-diphosphate (CPP) into copalol (**6**) were detected (Figure S1); (**B**) Total ion chromatograms (TIC) of control strain without diTPSs and the strain YS2 expressing codon optimized truncated *Tw*TPS9 and *Tw*TPS27; (**C**) Normalized peak areas to cell density of **1** and **2** for all yeast strains.

GC-MS analysis of the hexane-extracted cell pellets of yeast strains containing *Tw*TPS9 and *Tw*TPS27 in combination with a geranylgeranyl diphosphate synthase (GGPPS) confirmed formation of **1** and **2**, yet, and as in the transient system, predominant accumulation of the non-aromatized diterpenes **1** (Figure 3). This indicated that all generated variants of the diTPSs were functional in vivo. No diterpenes were produced by the empty vector control.

Additional peaks (Figure 3B) with retention time, mass over charge ratio, and fragmentation pattern matching hydrolyzed forms of the intermediates GGPP and CPP, respectively geranylgeraniol (GGOH) (**5**) and copalol (**6**), were also detected (Figure S1) as well as an unknown compound that co-eluted with **2** (trace amounts insuffiencient for identification, data not shown).

Strain YS2 (codon optimized truncated diTPS) showed the highest production capacity of both **1** and **2**. Normalized signal levels corresponding to **1** were 8.1- and 7.3-fold higher in YS2 compared to the lowest producing strains YS1 (codon optimized full length diTPS) and YS3 (full length native diTPS), respectively. Codon optimization of the full length diTPS did not significantly affect the ratio of **1** and **2** in YS1 compared to YS3 (Student *T*-test, *p* > 0.05). Truncating the diTPS to remove the putative chloroplast transit peptide (cTP) sequences in combination with codon optimization yielded a 2.8-fold higher accumulation of **1** in YS2 compared to YS4. The highest producing strain, YS2, was determined to yield 4.9 mg/L of **1**, which remains in the range of previously reported levels of diterpenes in engineered yeast [28,29,30].

All generated yeast strains formed hydrolyzation products of GGPP and copalyl diphosphate in the form of geranylgeraniol and copalol, respectively. This indicated that further optimized production could benefit from an increased activity of *Tw*TPS9 (producing CPP from GGPP) and *Tw*TPS27 (producing **1** from CPP), which emerged as limiting steps under the experimental conditions used.

### 2.3. Yeast In Vivo Production of Dehydroabietic Acid and Novel Acid Form of Miltiradiene

**Figure 4:**
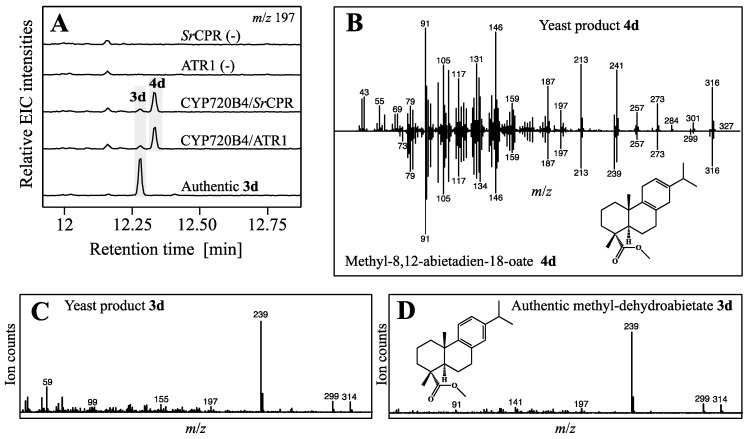
Production of the target and putative triptolide intermediate **3** and its analog **4** in yeast. (**A**) Extracted ion chromatograms (EIC) with *m*/*z* 197 of control strains expressing CPRs and diTPS, and strains additionally expressing *Ps*CYP720B4; (**B**) Comparison of MS spectra between yeast product **4d** (above) and reported spectrum of methyl-8,12-abietadien-18-oate (below) reported by [31]; (**C**) MS spectrum of yeast product **3d**; (**D**) MS spectrum of authentic methyl-dehydroabietate (**3d**) matching yeast product **3d**.

The highest-yielding strain YS2 was selected for co-expression of *Ps*CYP720B4, to test if further diterpene backbone modifications could be achieved. This also required testing of two NADPH dependent cytochrome P450 reductases (CPRs), ATR1 from *A. thaliana* and *Sr*CPR from *Stevia rebaudiana*, as potential electron donors. The introduction of *Ps*CYP720B4 and both CPRs independently into YS2 resulted in strains YS2-1 (*Ps*CYP720B4/ATR1) and YS2-2 (*Ps*CYP720B4/*Sr*CPR) and afforded **3d** and **4d** in TMSD derivatized ethyl acetate extracts from cell pellets analyzed by GC-MS (Figure 4A,B).

Compound **3d** showed the characteristic mass over charge ratio *m*/*z* of 314 (Figure 4C), fragmentation pattern and retention time identical to an authentic derivatized standard (Figure 4D). As in the transient expression system, levels of **2** were reduced significantly (Student *T*-test, *p* < 0.05) in strains expressing *Ps*CYP720B4 in combination with the CPRs ATR1 or *Sr*CPR, compared to the respective control strains, and consistent with efficient conversion into **3** derivatized to **3d** (Figure S2). This, to our knowledge, represents the first reported in vivo production of dehydroabietic acid from glucose in engineered yeast. As in the transient expression system, with a mass over charge ratio of *m*/*z* 316 (Figure 4B) and a match of the fragmentation pattern of 94% to spectra of previously reported methyl abieta-8,12-dien-18-oate [31], **4d** corresponds plausibly to the derivatized abieta-8,12-dien-18-oic acid (‘miltiradienic acid’) oxidized at carbon atom C-18 (Figure 4B). Levels of **1** displayed a similar significant drop (Student *T*-test, *p* < 0.05) in yeast strains expressing *Ps*CYP720B4 compared to the control strains further supporting high activity of the P450 (Figure S2). Aldehyde or hydroxylated intermediates were not detected in YS2-1 and YS2-2 under our experimental conditions (data not shown).

## 3. Discussion

Dehydroabietic acid (**3**), reported as metabolite from *T. wilfordii* cell cultures [22], is a key intermediate in proposed pathways towards triptolide. Our objective was to engineer production of **3** in yeast. Establishing a pathway in the transient system in tobacco required simultaneous co-expression of six heterologous enzymes together with the silencing suppressor P19: *Tw*TPS9 and *Tw*TPS27 in combination with *Ps*CYP720B4 and two enzymes from the upstream precursor pathway. The biosynthetic pathway was subsequently installed in an engineered yeast strain for production of **3** by heterologous expression of six biosynthetic genes (tHMGR1, *Sp*GGPPS7, *Tw*TPS9, *Tw*TPS27, *Ps*CYP720B4, and CPR). Detection of minor amounts of a novel compound, presumably corresponding to the acid analog of miltiradiene, **4** (‘miltiradienic acid’) is consistent with, and expands the earlier reported promiscuity of *Ps*CYP720B4, with a previously untested diterpene olefin. Aromatization of miltiradiene (**1**) to dehydroabietadiene (**2**) makes it plausible that **4** can also be oxidized to **3**, however this remains to be investigated as accumulation of **3** and **4** was not sufficient for quantification or oxidation assays.

The putative enzyme responsible for producing **3** in *T. wilfordii* cell cultures, or in the presumed biosynthetic route in planta has not yet been identified. Hence, to produce **3** in metabolically engineered yeast, alternative enzymes were considered. *Ps*CYP720B4 from Sitka spruce was chosen as candidate as it had previously been shown to catalyze most efficient in vitro full conversion of **2** into **3** [26]. Fully engineered integration of an active pathway in vivo for conversion in a heterologous host remained to be shown. Here, established microbial production from glucose of **3** via **2**, and furthermore demonstrated conversion of **1** to **4**, highlights the biotechnological impact of multifunctional (i.e., three-step) and promiscuous enzymes of the P450 family for functionalization of the inert hydrocarbon scaffold of diterpene olefins. Considering the immense structural diversity of labdane-type diterpene scaffolds, *Ps*CYP720B4 might represent an archetypal enzyme for installation of C-18 carboxylic acid functionality in other diterpenes.

A combination of codon optimization and N-terminal truncation of *Tw*TPS9 and *Tw*TPS27 allowed production of 4.9 mg/L **1** and its aromatized conversion product **2** in yeast. These findings support a long-standing notion [32] that substantially increased terpenoids production can be achieved by eliminating clusters of rare codons in the terpene synthases.

Truncated versions of the two diTPSs to remove putative chloroplast transit peptide (cTP) sequences were furthermore found to improve diterpene production. Chloroplast proteins such as diTPSs are typically translated in plants as pre-proteins carrying distinct chloroplast transit peptide (cTP) sequences. The sequences are required for their import into the chloroplasts before proteolytically cleaving to form mature proteins [33]. Removing the sequences coding for the cTP to produce ‘pseudomature’ proteins has previously been found to enhance activity, stability, and expression in *Escherichia coli* of diTPS [33,34]. Effects of truncating other diTPS producing **1** in yeast have previously not been investigated or reported [28,30]. However, a truncated class I diTPS (*Ro*KSL) from rosemary (*Rosmarinus officinalis*) forming **1** was not found functional in yeast, possibly due to incorrect prediction of the position [35]. It is plausible that other truncations of *Tw*TPS9 and *Tw*TPS27 could provide functional, and potentially even more efficient variants, as previous experiments with truncation-series indicated a range of activities [33,34].

Producing **2** in yeast at high yield is prerequisite for later sufficient push towards high-value compounds such as triptolide in biotechnological production. A detected misbalance between products generated by the diTPS in our system, and non-specific activities leading to hydrolysis of the diphosphate intermediates indicated a current limitation of the engineered pathway, the diTPS. Specifically, formation of CPP from the class II diTPS *Tw*TPS9 appeared consistently higher than turnover of CPP towards **1** by the class I diTPS *Tw*TPS27. Fine tuning the expression, and expression ratio of the class I to class II diTPS or identification of more efficient variants may help in addressing this issue.

Production of **3** by *Ps*CYP720B4 was confirmed in this study in vivo in yeast and the formation of **4** from **1** is suggested. Consistent with earlier reports [26], Sitka spruce *Ps*CYP720B4 produced fully oxidized **3** and here also of **4** from **1** and **2**, respectively. A mixture of C-18 and C-19 aldehyde or single hydroxylated diterpenoids was reported by the closely related homolog from loblolly pine CYP720B1 in yeast [18,30], which could suggest *Ps*CYP720B4 appears as a more efficient and regioselective choice over CYP720B1 for biotechnological production.

The yeast strain producing **3** in this study could potentially serve as a source for chemical synthesis of triptolide or triptolide derivatives [24,25]. However, **3** might even find direct applications as both **3** and its derivatives have been suggested to possess, among others, gastroprotective [36], anti-inflammatory [37] and anti-cancer properties [38]. The novel analog **4**, produced in this work and presumably representing abieta-8,12-dien-18-oic acid, might also have biological activities, but these remain to be explored pending improved production at higher levels.

The results presented in this work reveal potential targets for high impact optimization of diterpene backbone production in yeast. A functional diterpene pathway towards **3** was established, optimized for expression and exploited for production of oxidatively functionalized diterpenes from glucose in vivo in yeast.

## 4. Materials and Methods

### 4.1. Transient in Planta Expression in Tobacco

pLIFE33 vectors, derived from pCAMBIA1300 [39], harboring diterpene synthases (diTPS) from *Tripterygium wilfordii Tw*TPS9 (gene ID: 1041500503), *Tw*TPS27 (gene ID: 1041500481), *Ps*CYP720B4 from *Picea sitchensis* (Gene ID: 313756881), geranylgeranyl diphosphate synthase (*Cf*GGPPS) from *Coleus forskohlii* (Gene ID: 927521385), 1-deoxy-d-xylulose 5-phosphate synthase (*Cf*DXS) from *Coleus forskohlii* (Accesion number: KP889115), and tomato bushy shunt virus p19 protein (gene ID: 9790333) were transformed into *Agrobacterium* using previously described protocol [40]. Tobacco leaves were infiltrated and plants (Table S1) cultivated for six days in greenhouse conditions. Two leaf discs were used to extract produced diterpenes and samples were analyzed both derivatized and non-derivatized by GC-MS (Supplementary information).

### 4.2. Cloning and Construction of Yeast Plasmids

All PCR reactions were carried with iProof™ HF polymerase (Bio Rad) using standard protocol. Electrophoresis of PCR products was carried out on 1% (*w*/*v*) agarose gels and corresponding bands were cut out and purified using a Zymoclean™ Gel DNA recovery kit (Zymo Research) following standard protocol.

Dual expression vectors derived from the pSP-G series [41] were mainly used for gene assembly (Table S2) except single expression vector pEVE3. All plasmids used contained a CEN/ARS (centromere and autonomously replicating sequence).

Coding sequences from *T. wilfordii* encoding native diterpene synthases (diTPS) *TwTPS9* and *TwTPS27* were PCR amplified. Yeast codon optimized versions (Table S3) of *TwTPS9* and *TwTPS27*, designed to exclude the endonuclease sites HindIII, SacII, XhoI, SpeI, SacI, AarI, and PmeI were provided by GeneArt^®^ (Thermofisher Scientific, Zug, Switzerland).

Each diTPS was generated and tested in four variants: codon optimized, codon optimized truncated, native sequence, and truncated native sequence. To generate pseudomature variants of the enzymes, coding sequences were truncated to remove predicted chloroplast transit peptide (cTP) sequences (ChloroP 1.1 (available at http://www.cbs.dtu.dk/services/ChloroP/)). *Tw*TPS9 was predicted to contain a 46 amino acid cTP and *Tw*TPS27 a 41 amino acid cTP. *ATR1* (*Arabidopsis thaliana*, Gene ID: 3150037), *SrCPR* (*Stevia rebaudiana*, Gene ID: 93211213), and *SpGGPPS7* (*Synechococcus* sp., Gene ID: 86553638) were PCR amplified together with yeast codon optimized *PsCYP720B4* [26].

Genes were generally cloned by restriction/ligation cloning using enzymes from New England Biolabs (NEB, Whitby, ON, Canada). DNA blunt end reactions were carried out by DNA polymerase I LG (Klenow) large fragment (NEB). The blunt-ending reactions were carried out in CutSmart Buffer (New England Biolabs) with 0.01 mM dNTPs and 1 µL Klenow enzyme in 25 µL reaction volumes and were kept at 30 °C for 15 min followed by inactivation at 75 °C for 10 min. In-Fusion cloning (Takara Clontech, Saint-Germain-en-Laye, France) was carried out following standard protocol with constructs containing native diTPS due to internal endonuclease sites.

Cloned constructs were transformed into Mix and Go^®^ X10 Gold *Escherichia coli* competent cells (Zymo Research Europe GmbH, Feiburg im Breisgau, Germany) following standard protocol and plasmids were recovered using a ZR Plasmid Miniprep™—Classic (Zymo Research Europe GmbH) following included protocol. Purity and concentration were controlled by NanoDrop2000 (Thermofisher Scientific) and all constructs were sequence verified.

Yeast strains and transformation. The *S. cerevisiae* strain EYS2010 (MATalpha his3Δ1 leu2-3_112 ura3-52), carrying a truncated 3-hydroxy-3-methylglutaryl coenzyme A reductase (tHMGR1) in the YCT1 locus (Table 1) was utilized for all transformations. Yeast strain EYS2010 was grown overnight in 3 mL YPD media and inoculated into 50 mL YPD to an OD_600_ of 0.1. Transformations were carried out using standard lithium-acetate protocol as previously described [42].

### 4.3. Yeast Diterpene Production Procedure

In vivo production of diterpenes in yeast was carried out by incubating pre-cultures for 18–24 h in synthetic complete (SC) medium (6.7 g/L Yeast Nitrogen Base—Sigma Aldrich, Glucose 20 g/L, pH adjusted to 5.8) without the appropriate amino acids at 30 °C and adjustment of the OD_600_ to 0.1 of the main cultures in 25 mL selective SC medium in 250 mL shaking flasks. Cultures were incubated at 30 °C with 160 RPM for 72 h before final OD_600_ measurement, extraction, and chemical analysis.

### 4.4. Yeast Extraction Procedures

2 mL of the final yeast cultures was used for metabolite extraction and analysis: 2 mL culture was transferred to 2 mL screw cap microcentrifuge tubes and centrifuged at high speed (Centrifuge 5424, Eppendorf) for 4 min. Supernatants were discarded and pellets were dissolved in 1 mL 80% ethanol. The samples were then heated to 80 °C and agitated for 10 min followed by 4 min of high speed centrifugation. Supernatants were transferred to 14 mL glass vials and extracted: miltiradiene and dehydroabietadiene diterpenes were extracted with 1:1 n-hexane (AR, Biosolve, Dieuze, France) while DHA was extracted with 1:1 ethyl acetate (HPLC grade, Biosolve). Extracted samples were transferred to 1.5 mL glass vials after 10 seconds of vortexing to be ready for analysis. In the case of samples putatively containing DHA, 100 µL absolute methanol (HPLC grade, Biosolve) and 120 µL trimethylsilyl-diazomethane (TMSD in 2M diethyl ether, Sigma Aldrich, Buchs, Switzerland) were added for derivatization. The samples were then agitated at room temperature for 20 min prior to GC-MS analysis.

To verify identity and determine product levels, authentic and relative standards were used (dehydroabietic acid, derivatized as above for detection and quantification, Glentham Life Sciences, Corsham, England; *ent*-Kaurene, Evolva).

### 4.5. Analytical method

Extracted diterpenes from yeast strains were analyzed by an Agilent (Santa Clara, CA 95051, USA) 7890A/5975C GC-MSD with a HP-5MS column (Agilent, 30 m × 250 µm × 0.25 µm). The carrier gas helium was set at 1 mL·min^−1^ and 1 μL samples were injected in splitless mode at 250 °C. The GC oven temperature was programmed to start at 80 °C for 2 min, increase at 30 °C·min^−1^ from 80 °C to 170 °C, hold 170 °C for 3 min, increase at 30 °C min^−1^ from 170 °C to 300 °C and finally hold at 300 °C for 8 min. The total run-time was 20.33 min. MS detection was done in scan mode (mass-to-ion ratio, *m*/*z*, 35–500) with the EI source and the transfer line set at 250 °C and 280 °C, respectively.

Relative yield levels of diterpenes were calculated using the mean peak areas of extracted ion chromatograms (EIC) corresponding to miltiradiene (*m*/*z* 272) and dehydroabietadiene (*m*/*z* 255) normalized to OD_600_ values. Fragmentation patterns were compared and verified with existing patterns from the NIST14 Mass Spectral database, through both NIST 2.0 search and probability-based match (PBM) search, and by comparing with existing reported fragmentation patterns in literature.

## 5. Conclusions

Here we reported the identification of a biosynthetic route towards the putative triptolide precursor dehydroabietic acid by transient expression in *Nicotiana benthamiama* and scalable production from engineered *Saccharomyces cerevisiae*. We achieved efficient transient conversion of dehydroabietadiene to dehydroabietic acid and formation of a putative acid analogue, “miltiradienic acid”, from miltiradiene by expression of *Tripterygium wilfordii* diTPS *Tw*TPS9, *Tw*TPS27 and *Ps*CYP720B4 from Sitka Spruce. Our yeast strains could provide the basis towards elucidating the remaining triptolide pathway and furthermore as a scalable source of dehydroabietic acid and its analogue “miltiradienic acid”.

## Figures and Tables

**Table 1:** Yeast strains used and generated in this study.

Name	Genotype
EYS2010	*MATα*, *MAL2-8C*, *SUC2*, *his3Δ1*, *leu2-3_112*, *ura3-52*, *YCT1::P_GPD1_-tHMGR1-T_CYC1_*
YS1	[CEN/ARS/pPGK1-CO_*Tw*TPS9-tCYC1/URA3], [CEN/ARS/pTEF1-CO_*Tw*TPS27-tADH1/HIS3], [CEN/ARS/pCYC1-GGPPS7-tADH2/LEU2]
YS2	[CEN/ARS/pPGK1-CO_Δ46*Tw*TPS9-tCYC1/URA3], [CEN/ARS/pTEF1-COΔ41*Tw*TPS27-tADH1/HIS3], [CEN/ARS/pCYC1-GGPPS7-tADH2/LEU2]
YS3	[CEN/ARS/pPGK1-WT_*Tw*TPS9-tCYC1/URA3],[CEN/ARS/pTEF1-WT_*Tw*TPS27-tADH1/HIS3], [CEN/ARS/pCYC1-GGPPS7-tADH2/LEU2]
YS4	[CEN/ARS/pPGK1-WT_Δ46*Tw*TPS9-tCYC1/URA3], [CEN/ARS/pTEF1-WTΔ41*Tw*TPS27-tADH1/HIS3], [CEN/ARS/pCYC1-GGPPS7-tADH2/LEU2]
YS2-1	[CEN/ARS/pPGK1-CO_Δ46*Tw*TPS9-tCYC1/ pTEF1-COΔ41*Tw*TPS27-tADH11//URA3], [CEN/ARS/pPGK1-CO_*Ps*CYP720B4-tCYC1/pTEF1-ATR1-tADH11/HIS3], [CEN/ARS/pCYC1-GGPPS7-tCYC1/LEU2]
YS2-2	[CEN/ARS/pPGK1-CO_Δ46*Tw*TPS9-tCYC1/ pTEF1-COΔ41*Tw*TPS27-tADH11//URA3], [CEN/ARS/pPGK1-CO_*Ps*CYP720B4-tCYC1/pTEF1-ATR1-tADH11/HIS3], [CEN/ARS/pCYC1-GGPPS7-tCYC1/LEU2]
ATR1 (−)	[CEN/ARS/pPGK1-CO_Δ46*Tw*TPS9-tCYC1/pTEF1-COΔ41*Tw*TPS27-tADH11//URA3], [CEN/ARS/pTEF1-ATR1-tADH11/HIS3], [CEN/ARS/pCYC1-GGPPS7-tCYC1/LEU2]
*Sr*CPR (−)	[CEN/ARS/pPGK1-CO_Δ46*Tw*TPS9-tCYC1/ pTEF1-COΔ41*Tw*TPS27-tADH11//URA3], [CEN/ARS/pTEF1-*Sr*CPR-tADH11/HIS3], [CEN/ARS/pCYC1-GGPPS7-tCYC1/LEU2]

## Supplementary Materials

Supplementary materials are available online. Table S1: Tobacco plant combinations, Table S2: Yeast expression vectors, Figure S1: MS Spectra of yeast produced products. Figure S2: Diterpene production levels, Table S3: Yeast codon optimized sequences.
