# Supplementary material for: Production of Putative Diterpene Carboxylic Acid Intermediates of Triptolide in Yeast
Source: Molecules. 2017 Jun 13;22(6):981. doi: 10.3390/molecules22060981 (PMC6152743; doi:10.3390/molecules22060981)
Supplement: Supplementary file 1 [file molecules-22-00981-s001.pdf]

# Supplementary information

## Production of putative diterpene carboxylic acid intermediates of triptolide in yeast

**Victor Forman[1,2], Roberta Callari[2], Christophe Folly[2], Harald Heider[2] and Björn Hamberger[3,*]**

1 Evolva A/S, Lersø Park Allé 42-44, Copenhagen Ø, Denmark; victorf@evolva.com
2 Evolva Basel SA, Duggingerstrasse 23, Reinach, Switzerland; robertac@evolva.com
3 Michigan State University, Department of Biochemistry and Molecular Biology, 603 Wilson Road, East Lansing, MI 48824

* Correspondence: hamberge@msu.edu; Tel.: +1-517-884-6964

Academic Editor: name
Received: date; Accepted: date; Published: date

## Table of contents

Table S1
Table S2
Figure S1
Figure S2
Table S3

**TABLE S1**. Tobacco plant combinations.

| Name | Genes expressed | Putative heterologous diterpene products |
|---|---|---|
| Control | *Cf*GGPPS, *Cf*DXS, p19 | None |
| *Tw*TPS9+*Tw*TPS27 | *Cf*GGPPS, *Cf*DXS, p19, *Tw*TPS9, *Tw*TPS27 | dehydroabietadiene, miltiradiene |
| DiTPS+*Ps*CYP720B4 | *Cf*GGPPS, *Cf*DXS, p19, *Tw*TPS9, *Tw*TPS27, *Ps*CYP720B4 | Dehydroabietadiene, miltiradiene, dehydroabietic acid (DHA), "miltiradienic acid" (abieta-8,12-dien-18-oic acid) |

**TABLE S2.** Yeast expression vectors.

| Name | Promoter/Terminator | Yeast selection |
|---|---|---|
| pEVE1 | $P_{PGK1}/T_{CYC1}+P_{TEF1}/T_{ADH1}$ | *URA3* |
| pEVE2 | $P_{PGK1}/T_{CYC1}+P_{TEF1}/T_{ADH1}$ | *HIS3* |
| pEVE3 | $P_{CYC1}/T_{ADH2}$ | *LEU2* |

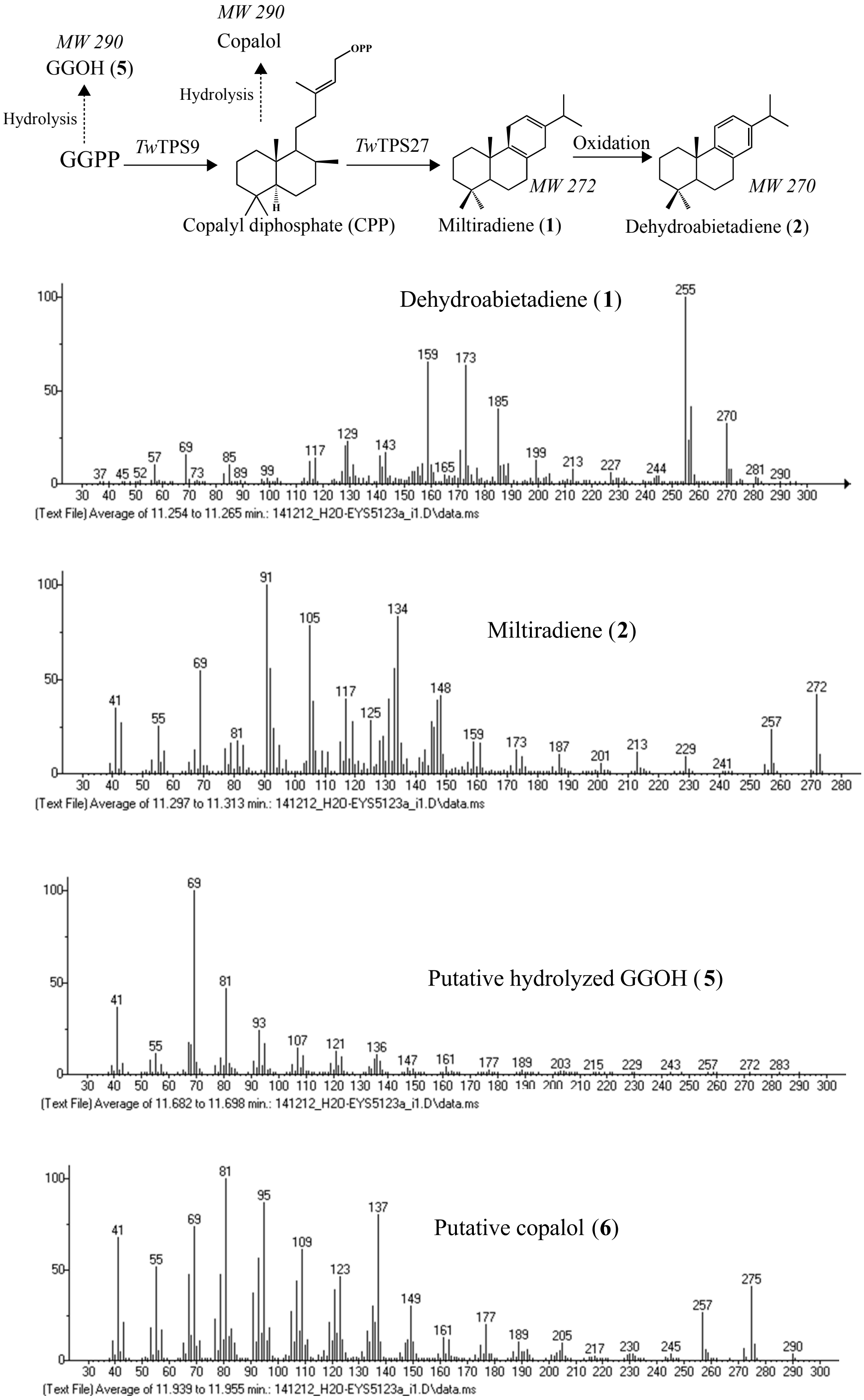


**Figure S1**. MS spectra and molecular weight (MW) of yeast produced products.

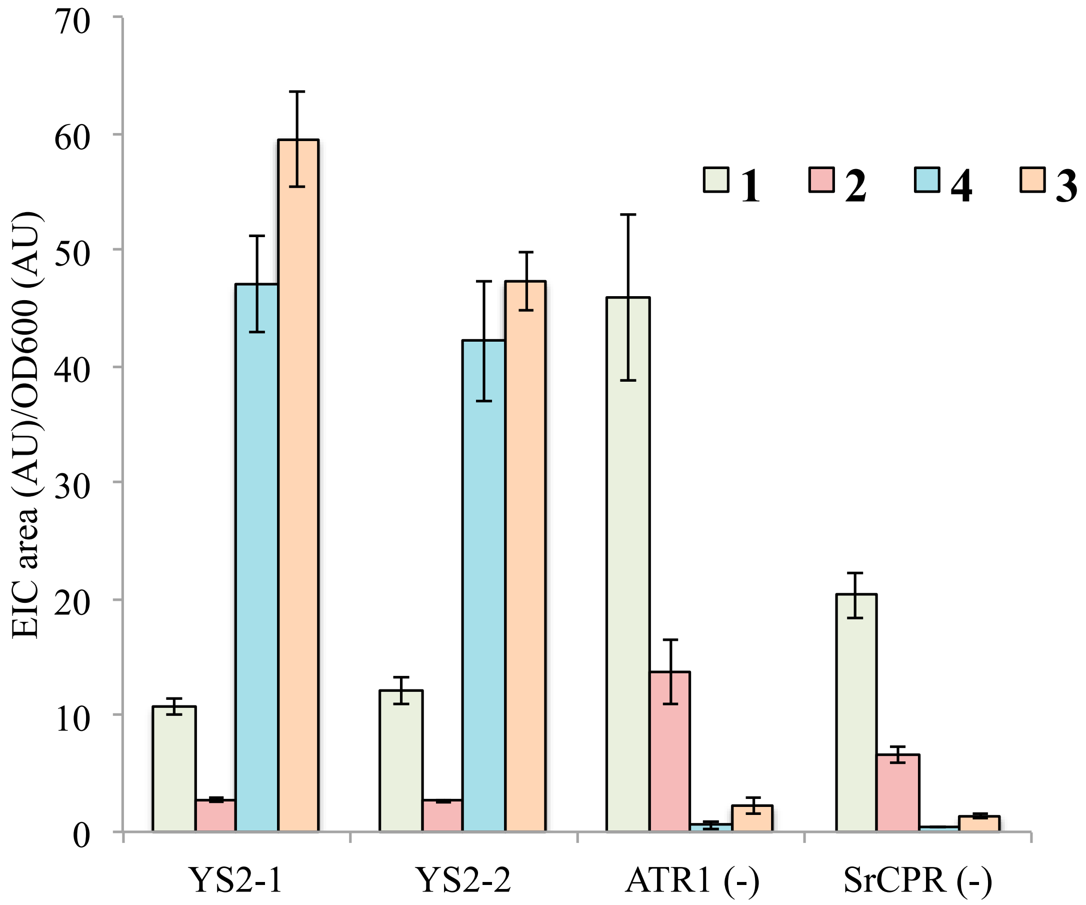

**Figure S2**. Diterpene production levels. Normalized levels (EIC area/$OD_{600}$) of miltiradiene (**1**), dehydroabietadiene (**2**), dehydroabietic acid (**3**) and miltiradienic acid (**4**) in yeast strains.

**TABLE S3.** Yeast codon optimized seqences.

| Name | Sequence |
|---|---|
| CO *Tw*TPS9 | ATGCACTCCTTGTTGATGAAGAAAGTCATCATGTACTCCTCCCAAACCACTCATGTTTTTCCATCTCCATTGCACTGTACTAT<br>CCCAAAGTCATCCTCATTTTTCTTGGATGCTCCAGTTGCTAGATTGCATTGCTTGTCTGGTCATGGTGCTAAAAAGAAGAGAT<br>TGCACTTCGATATCCAACAAGGTAGAAATGCTGTTTCTAAGACTCATACTCCAGATGACTTGTACGCTAAGCAAGAATACTCT<br>GTTCCAGAAATCGTTAAGGACGACGACAAAGAAGAAGAAGTCGTCAAGATCAAAGAACACGTTGACATCATCAAGTCCATGTT<br>GTCATCTATGGAAGATGGTGAAATTTCCATTTCCGCTTATGATACTGCTTGGGTTGCTTTGATTCAAGACATTCATAACAATG<br>GTGCTCCACAATTCCCATCATCTTTGTTGTGGATTGCCGAAAATCAATTGCCAGATGGTTCTTGGGGTGATTCTAGAGTTTTT<br>TTGGCTTTCGACAGAATCATTAACACCTTGGCTTGTGTTGTTGCCTTGAAGTCTTGGAATGTTCATCCAGATAAGTGCGAAAG<br>AGGTATCTCATTCTTGAAAGAAAACATCTCCATGTTGGAAAAGGACGACTCTGAACACATGTTGGTTGGTTTTGAATTTGGTT<br>TCCCAGTCTTGTTAGATATGGCTAGAAGATTGGGTATCGATGTTCCAGATGATTCTCCATTCTTACAAGAAATCTACGTCCAA<br>AGAGACTTGAAGTTGAAGAGAATCCCAAAGGATATCTTGCATAACGTTCCAACCACTTTGTTGCATTCCTTGGAAGCTATTCC<br>AGATTTGGATTGGACTAAGTTGTTGAAGTTGCAATGTCAAGACGGTTCCTTGTTGTTTTCTCCATCATCTACTGCTATGGCTT<br>TCATCAATACCAAGGACGAAAACTGCTTGAGATACTTGAATTACGTTGTCCAAAGATTCAATGGTGGTGCTCCTACTGTTTAT<br>CCATACGATTTGTTTGAACATAACTGGGCCGTTGACAGATTGCAAAGATTAGGTATTTCCAGATTCTTCCAACCAGAAATCAG<br>AGAATGTATGTCCTACGTTTACAGATACTGGACCAAGGATGGTATTTTCTGTACCAGAAACTCCAGAGTTCACGATGTTGATG<br>ATACAGCAATGGGTTTCAGATTATTGAGATTGCATGGTTACGAAGTTCACCCAGATGCTTTTAGACAATTCAAAAAGGGTTGC<br>GAATTCATCTGCTACGAAGGTCAATCTCATCCAACTGTTACTGTCATGTATAACTTGTACAGAGCCTCCCAATTGATGTTCCC<br>TGAAGAAAAGATTTTGGACGAAGCTAAGCAATTCACCGAAAAGTTTTTGGGTGAAAAGAGATCCGCTAACAAGTTGTTGGACA<br>AGTGGATTATTACCAAGGATTTGCCAGGTGAAGTTGGTTTCGCTTTGGATGTTCCATGGTATGCTTCATTGCCAAGAGTTGAA<br>GCTAGATTCTTCATCCAACATTACGGTGGTGAAGATGATGTTTGGTTGGATAAGGCATTATACAGAATGCCATACGTCAACAA<br>CAACGTCTATTTGGAATTGGCCAAGTTGGATTACAATTACTGTCAAGCCTTGCATAGAACTGAATGGGGTAGAATTCAAAAGT<br>GGTACGAAGAATGTAAGCCAAGAGATTTCGGTATCAGTAGAGAATGTTTGTTGAGAGCTTACTTTATGGCTGCTGCCTCTATT<br>TTTGAACCTGAAAGATCAATGGAAAGATTGGCTTGGGCTAAGACTGCTATTTTGTTGGAAATCATCGTTTCCTACTTCTCCGA<br>AGTTGGTAACTCTACCGAACAAAGAATTGCTTTCACTACCGAATTCTCTATTAGAGCTTCTCCAATGGGTGGTTACATTAACG<br>GTAGAAAATTGGACAAGATCGGTACTACCCAAGAATTGATCCAAATGTTGTTGGCTACCATCGACCAATTTTCACAAGATGCA<br>TTTGCTGCTTACGGTCATGATATTACTAGACACTTGCACAACTCATGGAAAATGTGGTTGTTGAAATGGCAAGAAGAAGGTGA<br>TAGATGGTTGGGTGAAGCCGAATTATTGATTCAAACCATTAACTTGATGGCCGATCATAAGATCGCTGAAAAGTTGTTTATGG<br>GTCACACCAACTACGAACAATTATTCTCTTTGACTAACAAGGTCTGCTACTCCTTGGGTCATCATGAATTGCAAAACAACAGA<br>GAATTGGAACACGATATGCAAAGATTGGTCCAATTGGTTTTGACCAACTCCTCTGATGGTATCGATTCCGATATTAAGAAAAC<br>CTTCTTGGCCGTTGCTAAGAGATTTTACTACACTGCTTTTGTTGATCCAGAAACCGTCAACGTTCATATTGCTAAGGTTTTGT<br>TCGAAAGAGTTGATTAA |
| CO *Tw*TPS27 | ATGGCTCCATTGGTTGTCTCCTTGACCATCTCCCATTTCGTTATTCAAACTGGTTCTACTGCCTTGCATTATTCTGCTTTGCC<br>AGAAACCAGAACTAAGCACTGTCATTCTTCTAGACCATTCGCCTCTATTAACTCCAACTCCTTGCAAATGAATCAAAGACCAT<br>TGACCGATTACAGACCAGCTATTTGGAATCCAGAATTGATCGATTCTTTGAACACCCCATACTCCTATCAATCTCATGGTACT<br>CAATTGGACAAGTTGAGACAAGATGCCAAGAGATTATTGTCCTCTACTTCTGATCCATGCTTGTTGTTGAACCACGTTGAATC<br>TATGCAAAGATTGGGTATTGCCTACCACTTCCAAGAAGAAATCGATTACTTGTTGAACACCAGAATCCAACCATACTCTCCAG<br>ATGATCATGACTTGCATACAACTGCTTTGAGATTCAGAATCTTGAGAGACAACAACTTCCCAATCTCCTCTGATGTTTTCGGT<br>AAGTTCATGTCCAGAGAAGGTAAATTCTTGGATTCTTTGTCTAGAGATGTCAAGGGTTTGTTGTCCTTGTATGAAGCTAGTTT<br>CTTGGGTGTTGATGGTGAAGTTATTTTGGACGAAGCCAAAGAATTCTCCTCAAAGAATTTGAGAGCTTTGTTGGGTAGATTGG<br>AATCAACCTCTATTGATGTTGCCGAACAAGTCAAGCAATCATTGCAAATTCCTTTGTTTTGGAGAATGCCTAGAGTTGAAGCC<br>AGAAACTTCATTGACTTCTACCAAAAGAAGGACGCCAAATCTTCTACCTTGTTGGAATTGGCTAAGTTGGATTTTAACTTGGT<br>CCAATCCACCTACCAACAAGAATTGAAAGAATTGTCTAAGTGGTGGGAAAACTTGGGTTTCAAGCAAAAGTTGTCTTTCACCA<br>GAGACAGATTGATGCAATCTTACTTCTCTACTACCGGTATTACCTTCAAGCCACAATTTTCCAAAGCTAGAATTGCTGCTACC<br>AAGTTCATCAACATCGTTAACACCATCGATGATATCCACGATTACTACGGTTCTCAAGATGACTTGAAGTTGTTTGATTCCGC<br>TGTTAAGAGATGGGATTTGGCTGCTATGGAAGAATTGCCAGATTACATGAAGATTTGCTACTTCGCCATGTACAACTTGGTTA<br>ACGAATTGGCTTACGACGTCTTGATCAATCAAGGTATAGATGTTTTGCCATGCTTGAGAGAAGCCTGGACTAAGTTTTGTGGT<br>GCTGCATTTGTTGAATCCCAATGGTGTTATACTGGTTACACTCCATCTATGGATGACTACTTGAAGAACTGCTGGATTTCCAT<br>TGGTGTTCACGGTTCTTTGAATTTTGCTAGAGCACATCAACAAGGTTCCAGATCTCCAATTGCTAATACTCCATTGCATTGCT<br>TGGAAGATCCTTTGTTGTATTGGTCCTCTGTCATCTGTAGATTGAACAACGATTTGGCTACCTTCCAACACGAATCTAAAACT<br>GGTGAAGTCGTTTCTTTCGTCAAGTGCTACATGGTTGAAAAGGGTGTTTCTCAAGAACAAGCCTGTGACGAAATTAGAGAATT<br>GATTAAGCACGCCTGGAAGATGTTGAATACCGAAAGAAGAAGATCTGACTTGCCACCATTGATGGTAGAAATGTGTATGGATA<br>CCCCAAAGTTGTCACAATGCTTGTATCAACATGGTGATGGTTTCGGTGTTGCTATTGATTTGACTAAGGATGTCATGTCCTCA<br>TTGATCTTCAGACAAATCCCAATTTGA |
